# Alterations in the human oral microbiome in cholangiocarcinoma

**DOI:** 10.1186/s40779-022-00423-x

**Published:** 2022-11-08

**Authors:** Ben-Chen Rao, Gui-Zhen Zhang, Ya-Wen Zou, Tong Ren, Hong-Yan Ren, Chao Liu, Zu-Jiang Yu, Zhi-Gang Ren

**Affiliations:** 1 Department of Infectious Diseases, the First Affiliated Hospital of Zhengzhou University, Zhengzhou, 450052 China; 2 Jinan Microecological Biomedicine Shandong Laboratory, Jinan, 250000 China; 3 Gene Hospital of Henan Province, Precision Medicine Center, the First Affiliated Hospital of Zhengzhou University, Zhengzhou, 450052 China; 4 Department of Pharmacy, the First Affiliated Hospital of Zhengzhou University, Zhengzhou, 450052 China; 5 Shanghai Mobio Biomedical Technology Co., Ltd., Shanghai, 201111 China

**Keywords:** Cholangiocarcinoma, Oral microbiome, Diagnostic biomarker, Random forest model, 16S rRNA MiSeq sequencing

Dear Editor,

Alterations in the human microbiome are closely related to various hepatobiliary diseases. Gut microbial dysbiosis has been found in patients with cholangiocarcinoma (CCA) [1]. However, the characteristics of oral microbiome in patients with CCA have not been studied.

Herein, a total of 272 saliva samples were prospectively collected. After the exclusion process, salivary samples from 74 patients with CCA, 150 healthy controls (HC) and 35 patients with hepatocellular carcinoma (HCC) were ultimately used for further analysis (Additional file 1). In the discovery phase, we characterized the CCA-associated microbiome and constructed a diagnostic model with 50 CCA patients and 100 HCs. Then, in the validation phase, the diagnostic model was validated by the other 24 CCA patients and 50 HCs. Finally, 35 HCC patients were used to evaluate the ability of the diagnostic model to distinguish intrahepatic cholangiocarcinoma (ICC) from HCC (Additional file 2: Fig. S1; Additional file 3: Table S1).

Compared with HC group, the platelets were significantly decreased, and liver function indices were worse in CCA group (Additional file 3: Tables S2, S3). The diversity analysis showed that the α-diversity and the abundance of rare species were significantly increased in CCA group than those in HC group (Fig. 1a; Additional file 2: Fig. S2a–c; Additional file 3: Tables S4, S5). The principal co-ordinates analysis (PCoA) (Fig. 1b) and nonmetric multidimensional scaling (NMDS) analysis (Additional file 2: Fig. S2d) indicated that the overall oral microbial composition was different between the two groups. Furthermore, a Venn diagram illustrated that 34 operational taxonomy units (OTUs) were exclusive to the CCA group (Fig. 1c). Subsequently, a heatmap based on the relative abundance of OTUs that had significant differences between the two groups showed that 6 OTUs including OTU17 (*Halomonas*), OTU74 (*Pelagibacterium*), OTU136 (*Prevotella*), OTU139 (*Prevotella*), OTU13 (*Peptostreptococcus*), and OTU18 ([*Eubacterium*]_nodatum group) were depleted in CCA group, and 60 OTUs, such as OTU30 (*Alloprevotella*), OTU61 (*Prevotella*) and OTU75 (*Alloprevotella*), OTU29 (*Neisseria*) and OTU119 (*Eikenella*) were enriched in the CCA group compared with the HC group (Additional file 2: Fig. S3; Additional file 3: Tables S6, S7).

**Fig. 1:**
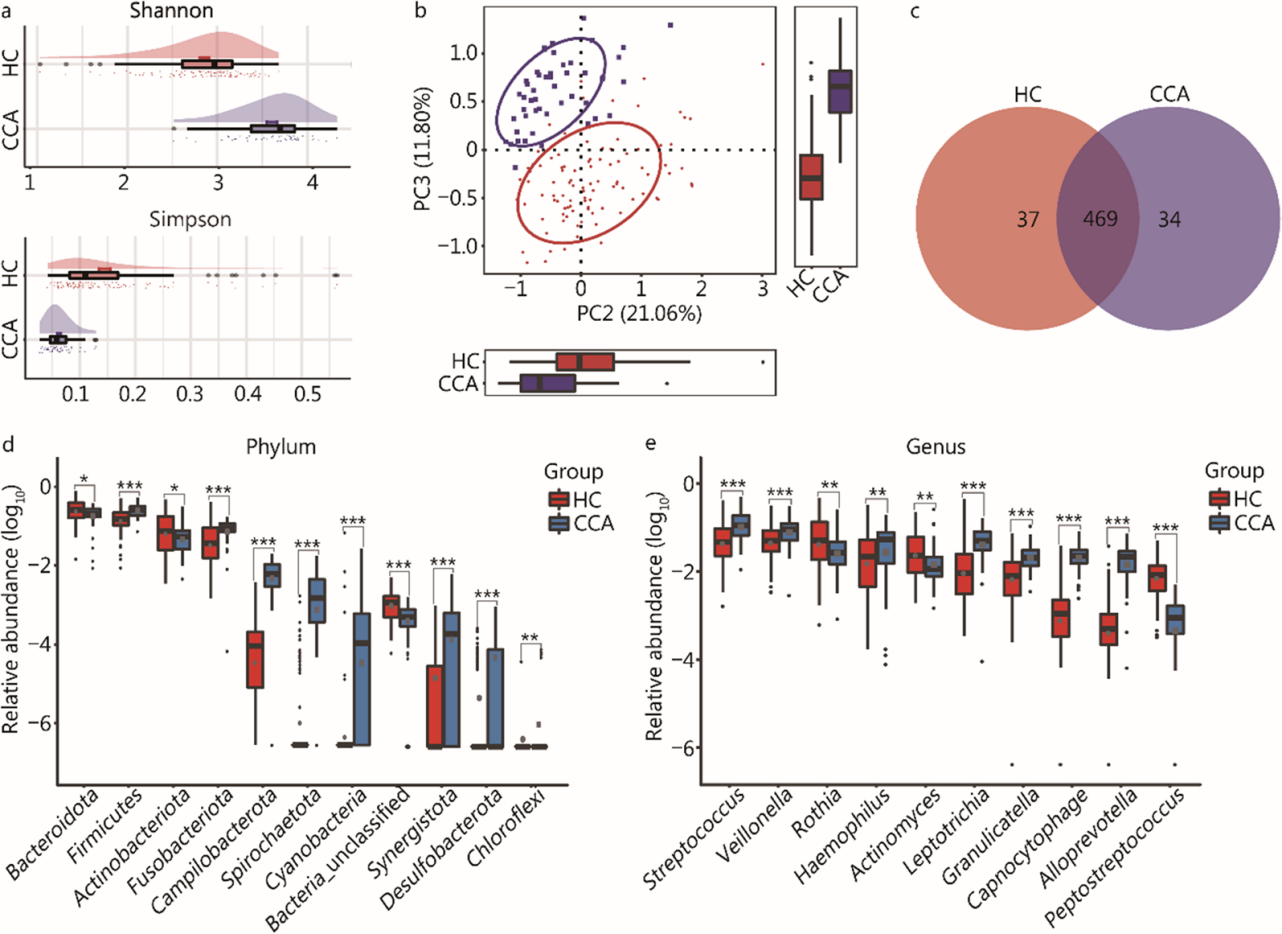
Characteristics of the oral microbiome in patients with CCA.** a** The Shannon index [(3.61 ± 0.05) vs. (2.86 ± 0.05), *P* < 0.001] and Simpson index [(0.06 ± 0.003) vs. (0.14 ± 0.01), *P* < 0.001] showed that the α-diversity of the oral microbial community was significantly increased in CCA group compared with HC group. **b** The PCoA showed that the samples of the CCA and HC groups were obviously separated in the direction of the PC2 axis and PC3 axis, showing that the overall oral microbial composition was different between the CCA and HC groups. **c** A Venn diagram based on microbial OTUs illustrated that 469 of the 540 OTUs were shared between the CCA group and HC group, and it is worth noting that 34 OTUs were exclusive to the CCA group. **d** At the phylum level, 8 phyla including *Firmicutes*, *Fusobacteriota*, *Campilobacterota*, *Spirochaetota*, *Cyanobacteria*, *Synergistota*, *Desulfobacterota* and *Chloroflexi* were significantly increased in CCA group, and 3 phyla covering *Bacteroidota*, *Actinobacteriota *and unclassified *Bacteria* were enriched in the HC group (*P* < 0.05). **e** At the genus level, 36 genera were identified as the genera with significant differences between the two groups (*P* < 0.05), the top 10 with the highest abundance were displayed, among which *Streptococcus*, *Veillonella*, *Haemophilus*, *Leptotrichia, Granulicatella, Capnocytophaga *and* Alloprevotella* were enriched in the CCA group, and *Rothia*, *Actinomyces* and *Peptostreptococcus* were enriched in the HC group. PCoA principal co-ordinates analysis, OTUs operational taxonomy units, CCA cholangiocarcinoma, HC healthy control

Then, we found that the compositions of the dominant species composition of the CCA and HC groups were similar (Fig. S2e–f). At the phylum level, 8 phyla which consisted of *Cyanobacteria*, *Spirochaetota*, *Campilobacterota*, *Fusobacteriota*, *Firmicutes*, *Synergistota*, *Desulfobacterota* and *Chloroflexi* were significantly increased in the CCA group, and 3 phyla, covering *Actinobacteriota*, *Bacteroidota* and unclassified *Bacteria* were enriched in the HC group (Fig. 1d; Additional file 3: Tables S8, S9). Moreover, at the genus level, 36 genera were identified as the genera with significant differences between the two groups (*P* < 0.05), the top 10 with the highest abundance were displayed in Fig. 1e, among which *Streptococcus*, *Veillonella*, *Haemophilus*, *Leptotrichia, Granulicatella, Capnocytophaga and Alloprevotella* were enriched in the CCA group, and *Rothia*, *Actinomyces* and *Peptostreptococcus* were enriched in the HC group (Additional file 3: Tables S10, S11). The phylogenetic characteristics and gene function of oral microbial communities were displayed in Additional file 2: Figs. S4–5 and Additional file 3: Tables S12–S14. Correlations between the microbiome and clinical characteristics were shown in Additional file 2: Fig. S6 and Additional file 3: Tables S15, S16).

The oral microbiome is used as a diagnostic biomarker in many diseases. However, the diagnostic potential of the oral microbiome for CCA has not been evaluated. Herein, we constructed a diagnostic model that could specifically identify CCA based on the oral microbiome. The fivefold cross-validation showed that the 3 OTU markers [(OTU20 (*Lautropia*), OTU30 (*Alloprevotella*) and OTU51 (*Actinomyces*)] were selected as the optimal marker set based on the discovery cohort (Additional file 2: Fig. S7a, b). We calculated the probability of disease (POD) index for each sample. In the discovery phase, the POD index was significantly increased in the CCA group (Additional file 2: Fig. S7c; Additional file 3: Table S17). The POD index achieved an AUC value of 0.9922 (Fig. S7d). To verify the diagnostic potential of the oral microbiome, the POD value was also significantly increased in the validation phase (Additional file 2: Fig. S7e; Additional file 3: Table S18) with a high AUC value of 0.9808 (Fig. S7f). Moreover, in clinical practice, ICC and HCC are often difficult to differentiate, so we tried to use the microbial diagnostic model to distinguish ICC from HCC. We redefined the POD index as the probability of ICC. The results showed that 18 ICC patients could be well distinguished from 35 HCC patients, the POD index was significantly increased in ICC group compared with HCC group (Additional file 2: Fig. S7g; Additional file 3: Table S19). The POD index achieved an AUC value of 0.9810 (Additional file 2: Fig. S7h).

Microbial dysbiosis has been reported in different parts of the human body in patients with CCA (Additional file 3: Table S20) [1–5]. Increased *Prevotella* was identified in the oral, gut and bile microbiome of patients with CCA [2, 5]. In addition, increased *Actinomyces* has been found in the gut and bile microbiome in CCA [1, 5]. However, in this study, the abundance of *Actinomyces* in oral cavity showed a significant decrease in CCA patients versus healthy individuals. Interactions between different human microbiomes in CCA patients need further research in the future. This study described the characteristics of the oral microbiome in CCA patients and reported the successful establishment of a diagnostic model of oral microbial markers for CCA. Moreover, oral microbiota-targeted biomarkers could serve as efficient and noninvasive diagnostic tools for CCA.

## Supplementary Information

**Additional file 1**. Consent informed and Supplementary methods.

**Additional file 2: Fig. S1**. Study design and flow diagram. **Fig. S2** Differences in the oral microbiome between the CCA and HC groups. **Fig. S3** A heatmap based on the relative abundance of OTUs which had significant difference between two groups. **Fig. S4** LEfSe and LDA analysis based on genera characterize microbiomes between the CCA and the HC groups. **Fig. S5** LDA scores predict gene function associated with oral microbiomes using PICRUSt [LDA Score (log_10_) > 3]. **Fig. S6.** Correlation in the microbiome and clinical characteristics. **Fig. S7** Noninvasive diagnostic model for CCA based on the oral microbiome.

**Additional file 3: Table S1**. Detailed reads and obtained OTUs for each individual sample. **Table S2** Clinical characteristics of participants in this study. **Table S3** Raw data of the clinical characteristics of participants in this study. **Table S4** Detail of the oral microbial α diversity index between the two groups. **Table S5** Raw data of the oral microbial α diversity index of each sample. **Table S6** Raw data of a heatmap based on the relative abundance of OTUs which had significant difference between two groups. **Table S7** A heatmap based on the relative abundance of OTUs which had significant difference between two groups. **Table S8** Comparison of the oral microbial community between the CCA and the HC groups at the phylum level. **Table S9** Relative abundance of the phyla between the two groups. **Table S10** Comparison of the oral microbial community between the CCA and the HC groups at the genus level. **Table S11** Relative abundance of the genera between the two groups. **Table S12** Cladogram using LEfSe method indicating the phylogenetic distribution of tongue coat microbes associated with patients with the CCA patients and the HC individuals. **Table S13** LDA scores showed the significant bacterial difference between the CCA and the HC groups at genus level [LDA Score (log_10_) > 3]. **Table S14** LDA scores predict gene function associated with oral microbiomes using PICRUSt [LDA Score (log_10_) > 3]. **Table S15** The *P*-value of Pearson correlation in the microbiome and clinical characteristics. **Table S16** Correlation coefficient of Pearson correlation in the microbiome and clinical characteristics. **Table S17** In the discovery phase, the POD index was significantly increased in the CCA group compared with that in the HC group. **Table S18** In the validation phase, the POD index was significantly increased in the CCA group compared with that in the HC group. **Table S19** The POD index was significantly increased in the ICC group compared with the HCC group. **Table S20** Alterations in the oral, gut and bile microbiome in patients with CCA.

## Data Availability

The raw Illumina read data for all samples were available through the European Nucleotide Archive (ENA) at the European Bioinformatics Institute (EBI) under accession number PRJNA846868.

## Abbreviations

AUC: Area under the curve
CCA: Cholangiocarcinoma
CI: Confidence interval
HC: Healthy control
HCC: Hepatocellular carcinoma
ICC: Intrahepatic cholangiocarcinoma
LDA: Linear discriminant analysis
NMDS: Nonmetric multidimensional scaling
OTUs: Operational taxonomy units
PCoA: Principal coordinate analysis
POD: Probability of disease
