# Supplementary material for: Alterations in the human oral microbiome in cholangiocarcinoma
Source: Mil Med Res. 2022 Nov 8;9:62. doi: 10.1186/s40779-022-00423-x (PMC9641929; doi:10.1186/s40779-022-00423-x)
Supplement: Supplementary file 1 — Additional file 1. Consent informed and Supplementary methods. [file 40779_2022_423_MOESM1_ESM.pdf]

Informed consent form for scientific research

(Translated from Chinese)

Dear participants,

We are from Department of Infectious Diseases, the First Affiliated Hospital of Zhengzhou University. We will free of charge help you monitor your health condition and record your clinical information and healthy/disease status or disease progression process. The collected saliva samples from participants in hospital will be used for scientific research. These results and data from the hospital electronic medical records will provide auxiliary data for clinical diagnosis and treatment, and will be used for scientific research. Thank you for your corporation.

Number:	Diagnosis:

The information that we collect from this research project will be kept confidential. Information about you that will be collected during the research will be put away and no-one but the researchers will be able to see it. Any information about you will have a number on it instead of your name. Only the researchers will know what your number is and we will lock that information up with a lock and key. It will not be shared with or given to anyone except our research team.

The knowledge that we get from doing this research will be shared with you through community meetings before it is made widely available to the public. Confidential information will not be shared. There will be small meetings in the community and these will be announced. After these meetings, we will publish the results in order that other interested people may learn from our research.

I have read the foregoing information, or it has been read to me. I have had the opportunity to ask questions about it and any questions that I have asked have been answered to my satisfaction. I consent voluntarily to participate as a participant in this research.

Print Name of Participant__________________

Signature of Participant ___________________

Date ___________________________

Day/month/year

A literate witness must sign (if possible, this person should be selected by the participant and should have no connection to the research team). Participants who are illiterate should include their thumb-print as well.

I have witnessed the accurate reading of the consent form to the potential participant, and the individual has had the opportunity to ask questions. I confirm that the individual has given consent freely.

Print name of witness_____________________

AND Thumb print of participant

Signature of witness ______________________

Date ________________________

Day/month/year

Statement by the researcher/person taking consent

I have accurately read out the information sheet to the potential participant, and to the best of my ability made sure that the participant understands that the following will be done:

1. We will free of charge help you monitor your health condition and record your clinical information and healthy/disease status or disease progression process.

2. These data from hospital electronic medical records will be used for scientific research.

3. The collected tongue coating, fecal, and serum samples will be used for scientific research.

I confirm that the participant was given an opportunity to ask questions about the study, and all the questions asked by the participant have been answered correctly and to the best of my ability. I confirm that the individual has not been coerced into giving consent, and the consent has been given freely and voluntarily.

A copy of this informed consent form has been provided to the participant.

Print Name of Researcher/person taking the consent________________________

Signature of Researcher /person taking the consent__________________________

Date ___________________________

Day/month/year

Supplementary methods

Study profile

The study was executed based on the prospective specimen collection and retrospective blinded evaluation design principles. The Ethics Committee of the First Affiliated Hospital of Zhengzhou University (2021-KY-0716-003) approved this study. Written informed consent was obtained from all participants.

A total of 272 saliva samples from Central China were prospectively collected. After a strict pathological diagnosis and exclusion process, salivary samples from 74 patients with CCA (including 18 intrahepatic cholangiocarcinoma (ICC), 42 hilar cholangiocarcinoma and 14 extrahepatic cholangiocarcinoma) and 150 healthy controls (HC) were subjected to 16S rRNA MiSeq sequencing. Moreover, 35 samples from HCC patients were collected to evaluate the ability of the diagnostic model to distinguish ICC from HCC. All enrolled patients with CCA or HCC were diagnosed based on the pathological findings of surgically resected tissues. The demographics and clinicopathological data of the participants were collected from hospital electronic medical records and questionnaires.

All saliva samples originated from newly diagnosed CCA and HCC patients from the First Affiliated Hospital of Zhengzhou University. All enrolled patients were diagnosed by clinicopathological data. All participants who had the following diseases were excluded: all types of diseases in the oral cavity, other tumors, diabetes, metabolic syndrome, and nonalcoholic fatty liver disease. In addition, all participants who received antibiotics and/or probiotics within 8 weeks before providing samples were also excluded.

Saliva sample collection

Saliva samples were provided by each participant between 7 am and 9 am. Saliva samples were collected as described in our previous study [1]. On the day of sampling, the participants were asked

to eat and brush their teeth after providing saliva samples. The participants were requested to rinse their mouths twice with sterile water before providing saliva samples. A total of 5 ml saliva rather than phlegm was asked to be spat into the sterile saliva collection tube. All the samples were stored at -80 °C as soon as possible, and we excluded the samples that were at room temperature for more than 2 hours.

DNA extraction

The microbial DNA in saliva samples were extracted by the Qiagen Mini Kit (Qiagen, Hilden, Germany) as described previously [2]. The samples were processed by phenol trichloromethane DNA extraction using a bead beater to mechanically disrupt cells, followed by phenol-chloroform extraction. Then, the DNA was purified in accordance with the manufacturer's instructions. The DNAs were quantified by the Qubit 2.0 Fluorometer (Invitrogen, Carlsbad, CA, USA), and molecular size was estimated using agarose gel electrophoresis. All microbial DNAs were diluted to 10 ng/μl for microbial analysis.

PCR amplification

The details of PCR amplification were performed according to our previous study [2]. PCR primers incorporated sample-specific barcodes for multiplex sequencing using the Illumina MiSeq System (paired-end 250-nt reads). The extracted DNA samples were amplified by primers (5'-ACTCCTACGGGAGGCAGCA-3' and 5'-GGACTACHVGGGTWTCTAAT-3') which targeted the hypervariable V3-V5 region of the 16S rRNA gene. The PCR reaction system contained 10 ng of template DNA, 0.8 μl of each primer (5 μmol/L), 0.8 μl of each primer (5 μmol/L), 0.4 μl of Fast PFU polymerase,4 μl of 5×Fast PFU buffer and 2 μl of 2.5 mmol/L dNTP (TransGen Biotech, Beijing, China). Four PCR reactions were conducted on each sample in a PCR machine (ABI GeneAmp 9700) as follows: 95 °C for 2 min, 95 °C for 30 s, 55 °C for 30 s, 72 °C for 30 s, repeating 30 cycles and finally at 72 °C for 5 min. Pool the PCR products from the same sample. Separate, extract and purify

the PCR products by Agarose gel (Axygen Biosciences, Union City, CA). At last, we used a fluorescence assay kit (Quant-iT PicoGreen, Invitrogen) to quantify the products.

MiSeq sequencing and data processing

Sequencing was accomplished on an Illumina MiSeq platform by Shanghai Mobio Biomedical Technology, China [2]. A negative control was used in the PCR amplification process. The details of PCR amplification, sequencing and sequence data processing can be found in the Supplementary Methods. The raw Illumina read data for all samples were uploaded to the European Nucleotide Archive (ENA) at the European Bioinformatics Institute (EBI) under accession number PRJNA846868.

The amplified reads were performed in accordance with the following steps: (a) use the FLASH v1.2.10 software to overlap pair end sequenced reads. (b) use the customization of each program to conduct more specific quality control on overlapping reads generated by FLASH: 1) Exclude no ambiguous bases in reads; 2) Exclude the samples whose mismatch rate in the overlapping area were over 0.05; 3) Exclude 0o mismatches in the primer and barcode region. (c) de-multiplex and assign reads into different samples according to the barcodes; (d) the chimeric sequences were detected and removed by UCHIME version 4.2.40 (version microbiome util-r20110519, http://drive5.com/uchime/gold.fa) to match Operational Taxonomy Units (OTUs).

Operational taxonomy unit (OTU) clustering and taxonomy annotation

All samples with equal numbers were selected for random reads, and the OTUs were binned by the UPARSE pipeline. We set the identity threshold at 0.97. RDP classifier version 2.6 [3] was used and the confidence level was set as 0.5 to annotate sequences [4]. The processes of microbial diversity and taxonomic analysis are provided in the Supplementary Methods.

Microbial diversity and taxonomic analysis

The microbial diversity was calculated based on the sampling OTU analysis. The diversity indexes

including Simpson index and Shannon index were calculated by the R software by the ‘vegan’ package. Principal coordinate analysis (PCoA) was conducted by the R package (http://www.R-project.org/) [5]. The Phyloseq package was used to calculate the weighted and unweighted unifrac distances. The P value < 0.05 was set for the key variables in constructing the heatmap by the Heatmap Builder.

Gene function prediction

PICRUSt was used to predict the gene function and KEGG pathways for the oral microbiome between the CCA and HC groups [6]. Considering the relative differences in the copy number of 16S rRNA genes between species, the abundance data of the original species were corrected to conduct a more accurate prediction process.

Identification of OTU biomarkers and construction of probability of disease (POD) index

The POD index was constructed according to our previous study [7]. We selected the OTU biomarkers by the Wilcoxon rank-sum test. A fivefold cross-validation was performed on a random forest model by the abundance profile of the optimal OTU markers (R 3.4.1, randomForest 4.6-12 package). The cross-validation error curve was calculated through five trials of the fivefold cross validation. We defined the point with the minimum cross-validation error as the cutoff point through the minimum error plus the standard deviation (SD). The optimal OTUs were defined by the smallest number of OTU sets with an error less than the cutoff value. The POD index was calculated by the optimal set of OTUs. Furthermore, the receiver operating characteristic (ROC) curve was used to assess the model by the R package pROC.

Statistical analysis

Statistical analyses were performed using SPSS V.20.0 for Windows (SPSS, Chicago, Illinois, USA). Categorical variables between two groups were compared by the χ^2 test or Fisher’s exact test. Continuous variables between two groups were compared by Student’s t test or Wilcoxon rank-sum

test. We defined a *P*-value < 0.05 (two-sided) as significant.

References

1. Rao B, Lou J, Lu H, Liang H, Li J, Zhou H, et al. Oral microbiome characteristics in patients with autoimmune hepatitis. Front Cell Infect Microbiol. 2021; 11:656674.
2. Ren Z, Wang H, Cui G, Lu H, Wang L, Luo H, et al. Alterations in the human oral and gut microbiomes and lipidomics in COVID-19. Gut. 2021; 70(7):1253-65.
3. Edgar RC. UPARSE: highly accurate OTU sequences from microbial amplicon reads. Nat Methods. 2013; 10(10):996-8.
4. Wang Q, Garrity GM, Tiedje JM, Cole JR. Naive Bayesian classifier for rapid assignment of rRNA sequences into the new bacterial taxonomy. Appl Environ Microbiol. 2007; 73(16):5261-7.
5. McMurdie PJ, Holmes S. phyloseq: an R package for reproducible interactive analysis and graphics of microbiome census data. PLoS One. 2013; 8(4):e61217.
6. Lu H, Ren Z, Li A, Zhang H, Jiang J, Xu S, et al. Deep sequencing reveals microbiota dysbiosis of tongue coat in patients with liver carcinoma. Sci Rep. 2016; 6:33142.
7. Ren Z, Li A, Jiang J, Zhou L, Yu Z, Lu H, et al. Gut microbiome analysis as a tool towards targeted non-invasive biomarkers for early hepatocellular carcinoma. Gut. 2019; 68(6):1014-23.
8. Jia X, Lu S, Zeng Z, Liu Q, Dong Z, Chen Y, et al. Characterization of gut microbiota, bile acid metabolism, and cytokines in intrahepatic cholangiocarcinoma. Hepatology. 2020; 71(3):893-906.
9. Deng T, Li J, He B, Chen B, Liu F, Chen Z, et al. Gut microbiome alteration as a diagnostic tool and associated with inflammatory response marker in primary liver cancer. Hepatol Int. 2022;16(1):99-111.
10. Saab M, Mestivier D, Sohrabi M, Rodriguez C, Khonsari MR, Faraji A, et al. Characterization of biliary microbiota dysbiosis in extrahepatic cholangiocarcinoma. PLoS One. 2021;16(3):e0247798.
11. Chen B, Fu SW, Lu L, Zhao H. A preliminary study of biliary microbiota in patients with bile duct stones or distal cholangiocarcinoma. Biomed Res Int. 2019; 2019:1092563.
12. Li Z, Chu J, Su F, Ding X, Zhang Y, Dou L, et al. Characteristics of bile microbiota in cholelithiasis, perihilar cholangiocarcinoma, distal cholangiocarcinoma, and pancreatic cancer. Am J Transl Res. 2022;14(5):2962-71.
