# Supplementary material for: Alterations in the human oral microbiome in cholangiocarcinoma
Source: Mil Med Res. 2022 Nov 8;9:62. doi: 10.1186/s40779-022-00423-x (PMC9641929; doi:10.1186/s40779-022-00423-x)
Supplement: Supplementary file 2 — Additional file 2: Fig. S1. Study design and flow diagram. Fig. S2 Differences in the oral microbiome between the CCA and HC groups. Fig. S3 A heatmap based on the relative abundance of OTUs which had significant difference between two groups. Fig. S4 LEfSe and LDA analysis based on genera characterize microbiomes between the CCA and the HC groups. Fig. S5 LDA scores predict gene function associated with oral microbiomes using PICRUSt [LDA Score (log10) > 3]. Fig. S6. Correlation in the microbiome and clinical characteristics. Fig. S7 Noninvasive diagnostic model for CCA based on the oral microbiome. [file 40779_2022_423_MOESM2_ESM.pdf]

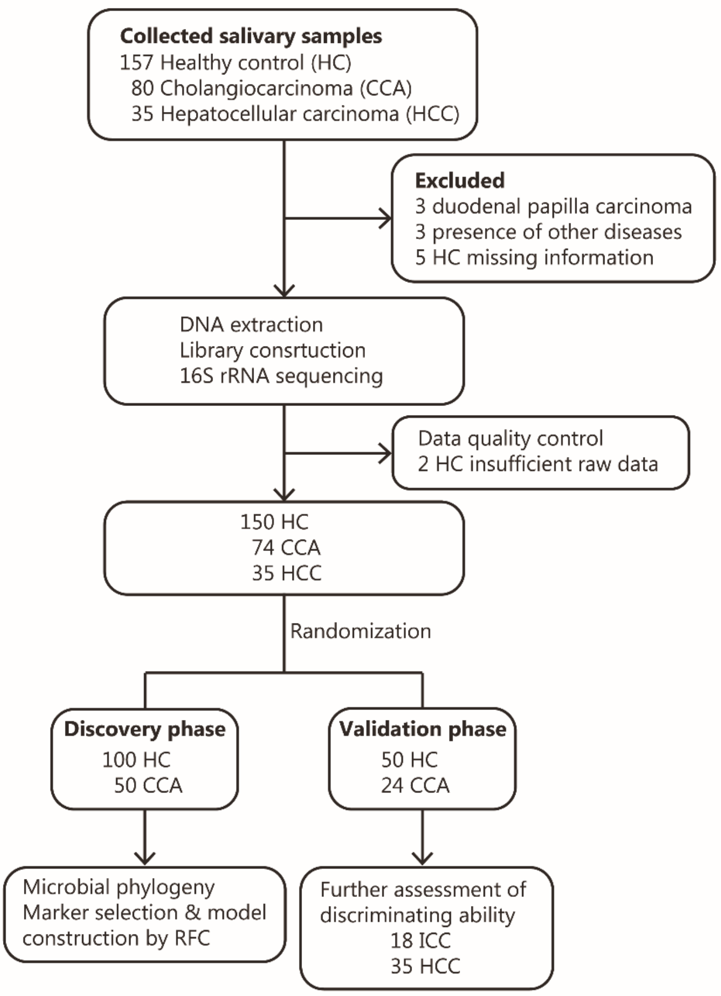


**Fig. S1 Study design and flow diagram**. A total of 272 saliva samples from Central China were prospectively collected. After a strict pathological diagnosis and exclusion process, salivary samples from 74 patients with CCA, 150 HC and 35 patients with HCC were ultimately used for subsequent analyses. In the discovery phase, we characterized the CCA-associated microbiome and constructed a diagnostic model using 50 CCA patients and 100 HC. Then, the model was validated by the other 24 CCA patients and 50 HC. Finally, 35 samples from HCC patients were collected to evaluate the ability of the diagnostic model to distinguish ICC from HCC. CCA cholangiocarcinoma, HC healthy control, RFC random forest model, ICC intrahepatic cholangiocarcinoma, HCC hepatocellular carcinoma

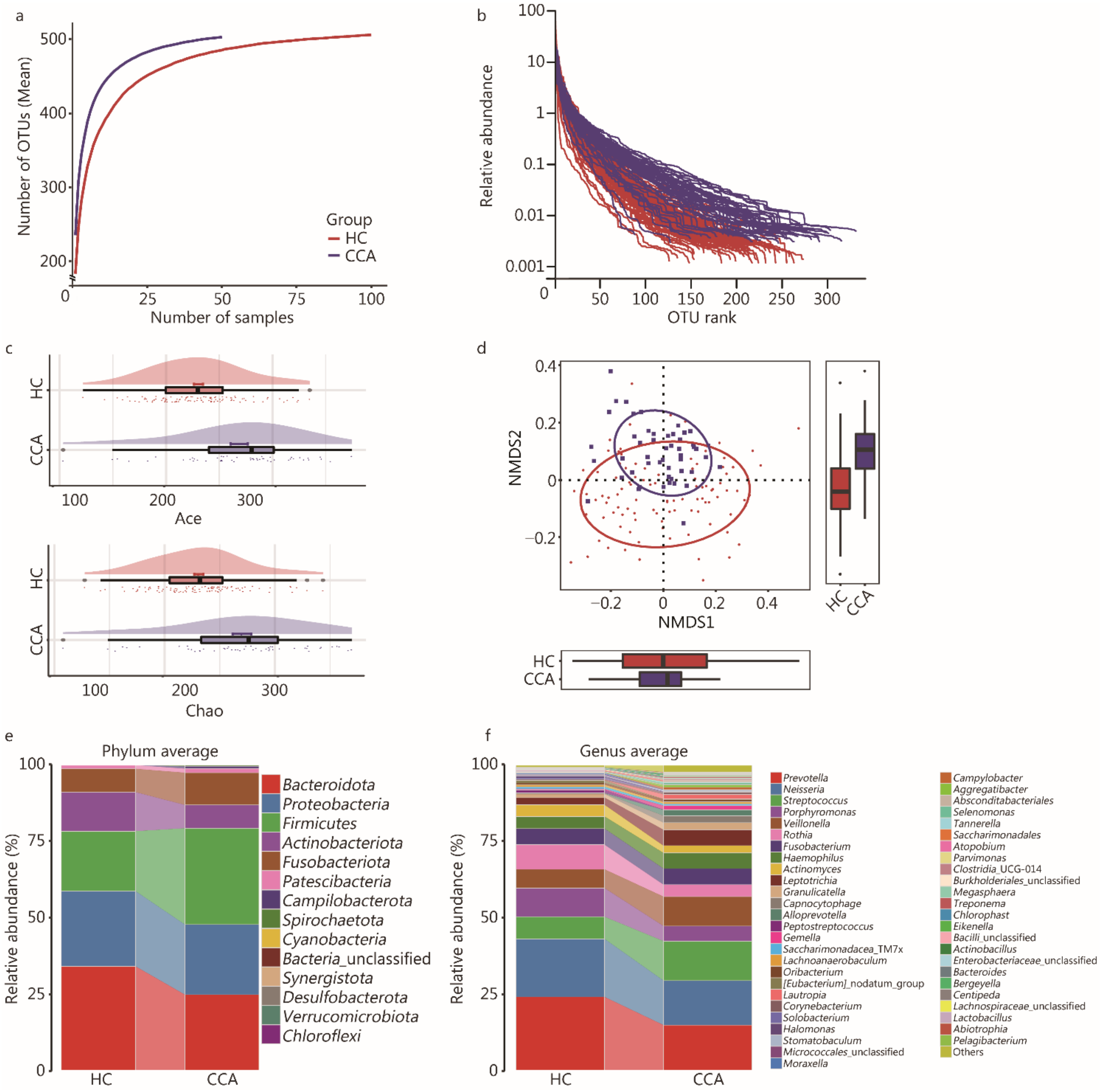


**Fig. S2 Differences in the oral microbiome between the CCA and HC groups. a** Rarefaction analysis of the oral microbiome showed that as the number of samples increased, the slope of the curve gradually approached 0 which means the sample size of both groups was appropriate to a certain extent. **b** Rank-abundance curve showed that the species richness and evenness of CCA group were significantly higher than those of HC group. **c** Abundance of rare species was significantly increased in CCA group compared with HC group according to the Ace index [(268.78 ± 7.97) vs. (230.48 ± 4.21), $P < 0.001$] and the Chao index [(270.21 ± 8.50) vs. (231.03 ± 4.14), $P < 0.001$]. **d** The NMDS analysis showed that the samples of the CCA and HC groups were obviously separated in the direction of the NMDS1 axis and NMDS2 axis, showing that the overall oral microbial composition was different between the CCA and HC groups. **e** At the phylum level, we found that the dominant species

compositions of the CCA and HC groups were similar. The *Fusobacteriota*, *Actinobacteriota*, *Firmicutes*, *Proteobacteria* and *Bacteroidota* accounted for more than 90% of all phyla. **f** At the genus level, *Porphyromonas*, *Streptococcus*, *Neisseria*, *Prevotella* and *Veillonella* were the most abundant genera, and these genera made up 60% of all genera. OTUs operational taxonomy units, NMDS nonmetric multidimensional scaling, CCA cholangiocarcinoma, HC healthy control

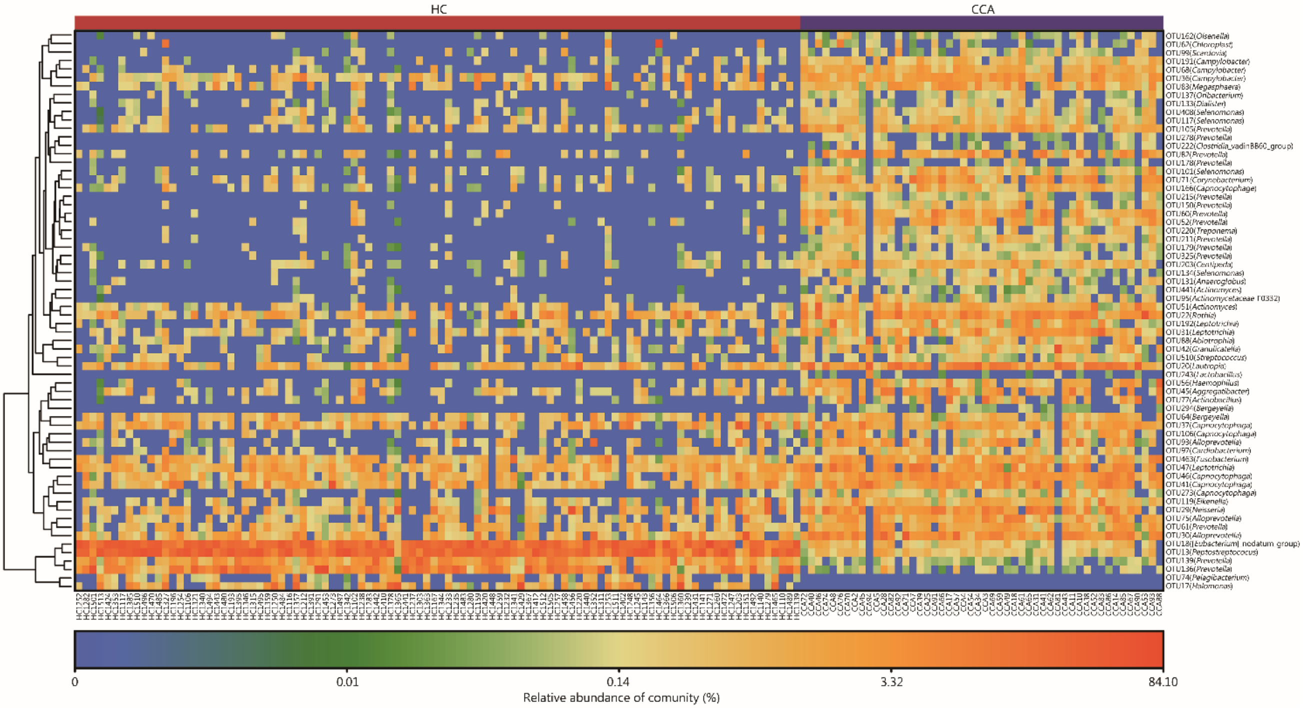


**Fig. S3 A heatmap based on the relative abundance of OTUs which had significant difference between two groups.** The result showed that 6 OTUs which consisted of OTU17 (*Halomonas*), OTU74 (*Pelagibacterium*), OTU136 (*Prevotella*), OTU139 (*Prevotella*), OTU13 (*Peptostreptococcus*), and OTU18 ([*Eubacterium*]_nodatum group) were depleted in CCA group, and 60 OTUs, such as OTU30 (*Alloprevotella*), OTU61 (*Prevotella*) and OTU75 (*Alloprevotella*), OTU29 (*Neisseria*) and OTU119 (*Eikenella*) were enriched in the CCA group compared with the HC group. OTUs operational taxonomy units, CCA cholangiocarcinoma, HC healthy control

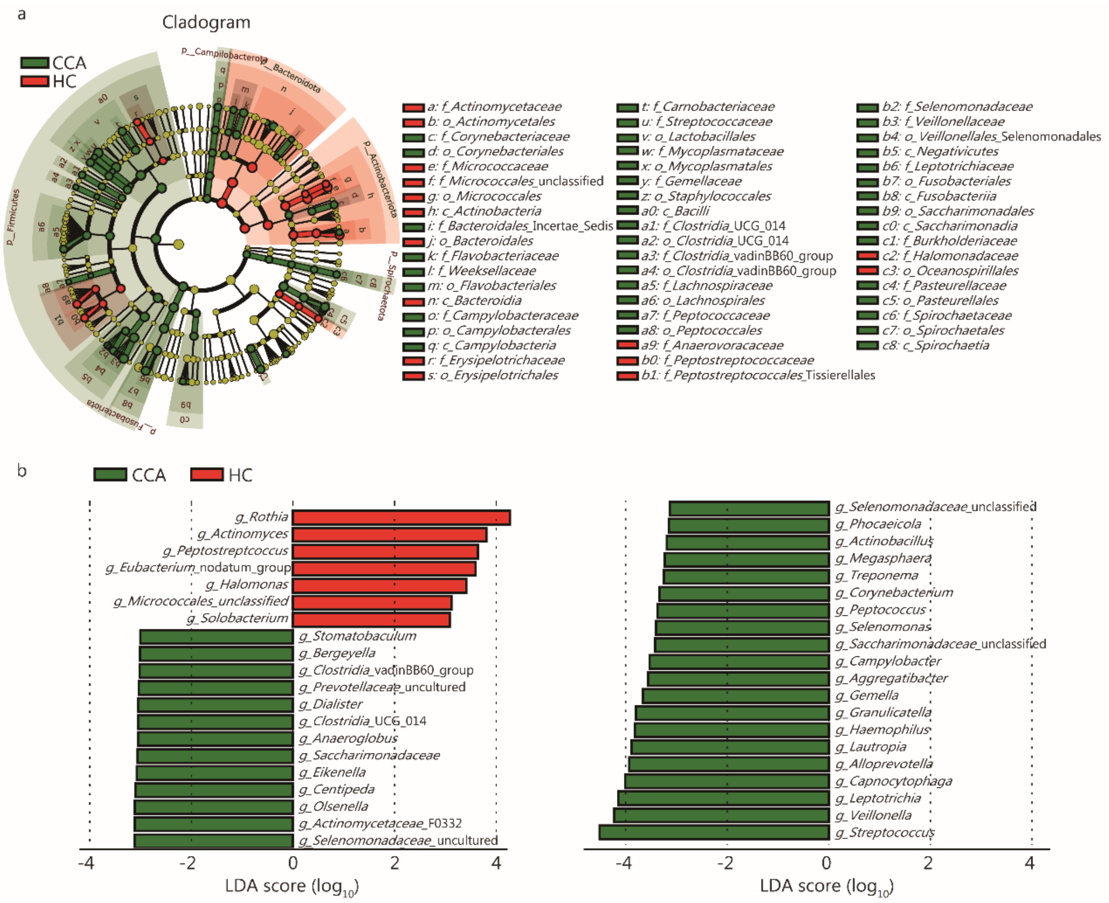


**Fig. S4 LEfSe and LDA analysis based on genera characterize microbiomes between the CCA and the HC groups. a** Cladogram using LEfSe method indicating the phylogenetic distribution of tongue coat microbes associated with patients with the CCA and HC groups. **b** Oral microbiome in CCAs was characterized by a preponderance of *Streptococcus*, *Veillonella*, *Leptotrichia*, *Capnocytophaga* and *Alloprevotella* at the genus level [LDA Score $(\log_{10}) > 3$], whereas the HC microbiome was characterized by a preponderance of *Rothia*, *Actinomyces*, *Peptostreptococcus*, *Eubacterium nodatum* group and *Halomonas*. LEfSe linear discriminant analysis effect size, LDA linear discriminant analysis, CCA cholangiocarcinoma, HC healthy control

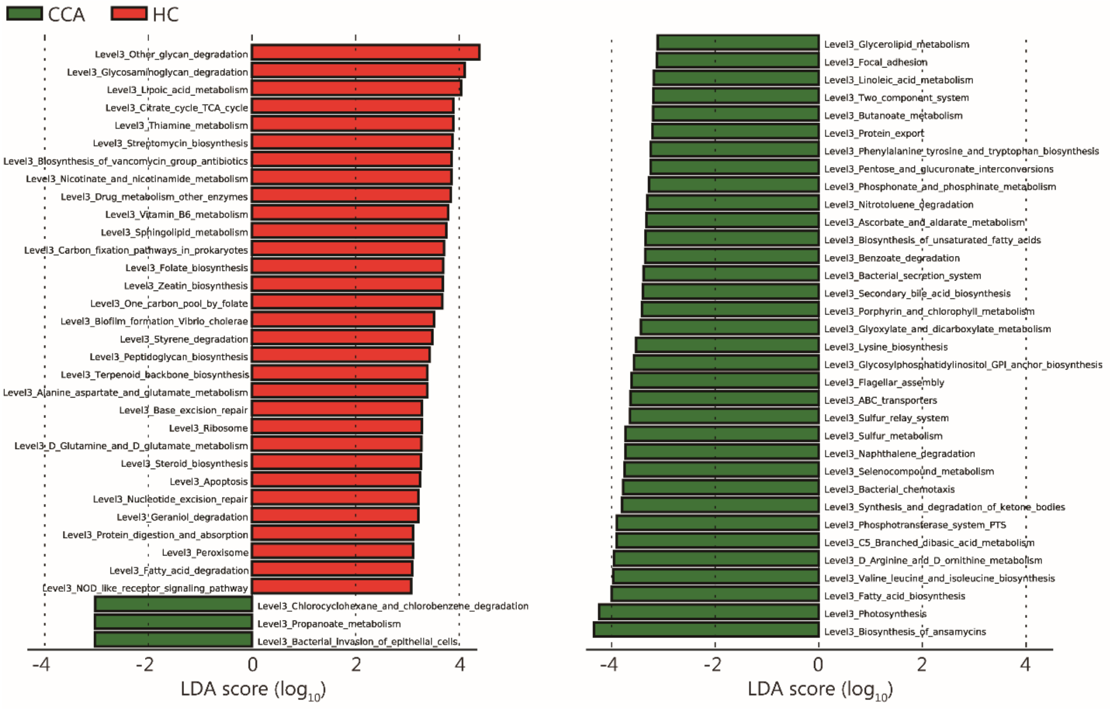


**Fig. S5 LDA scores predict gene function associated with oral microbiomes using PICRUSt [LDA Score ($log_{10}$) > 3].** The enriched Kyoto Encyclopedia of Genes and Genomes (KEGG) pathways were identified by PICRUST software at Level 3 [LDA Score ($log_{10}$) > 3]. The results showed that many metabolic pathways were enriched in the CCA group, these pathways included, the biosynthesis processes of ansamycins, fatty acids, valine leucine and isoleucine and the metabolic processes of arginine, ornithine, branched dibasic acid and some other transporters. LDA linear discriminant analysis, PICRUSt Phylogenetic Investigation of Communities by Reconstruction of Unobserved States, CCA cholangiocarcinoma, HC healthy control

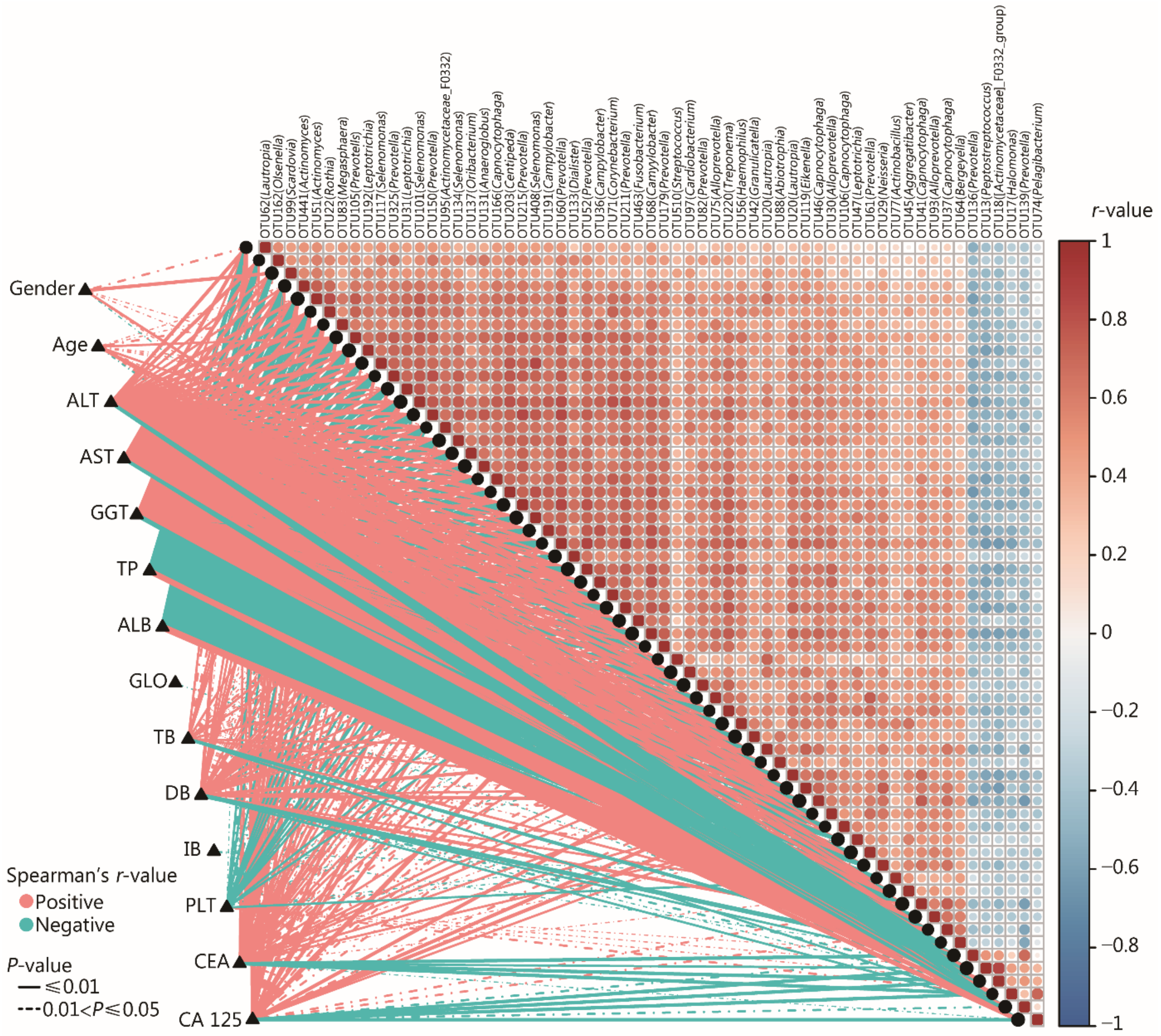


**Fig. S6. Correlation in the microbiome and clinical characteristics.** Spearman's correlation analysis showed the correlation between 61 OTUs and 14 clinical indices. The results indicated that OTU74 (*Pelagibacterium*) was negatively correlated with age, ALT, AST, GGT, CEA and CA125, and positively correlated with TP and ALB. Additionally, OTU139 (*Prevotella*) was negatively correlated with TB, DB and CEA. In addition, CEA was negatively correlated with 5 OTUs (OTU17 (*Halomonas*), OTU18 ([*Eubacterium*]_nodatum_group), OTU136 (*Prevotella*), OTU13 (*Peptostreptococcus*) and OTU74 (*Pelagibacterium*)) and positively correlated with 42 OTUs including OTU20 (*Lautropia*), OTU22 (*Rothia*), OTU31 (*Leptotrichia*), OTU36 (*Campylobacter*) and OTU42 (*Granulicatella*). The above results suggest that there was a close relationship between oral microbiome and clinical indicators. OTUs operational taxonomy units, PLT platelets, ALT alanine aminotransferase, AST aspartate aminotransferase, GGT gamma-glutamyltransferase, TP total protein, ALB albumin, GLO globulin, TB total bilirubin, DB direct bilirubin, IB indirect bilirubin, CEA carcinoembryonic antigen, CA125 carbohydrate antigen125, CCA cholangiocarcinoma, HC healthy control

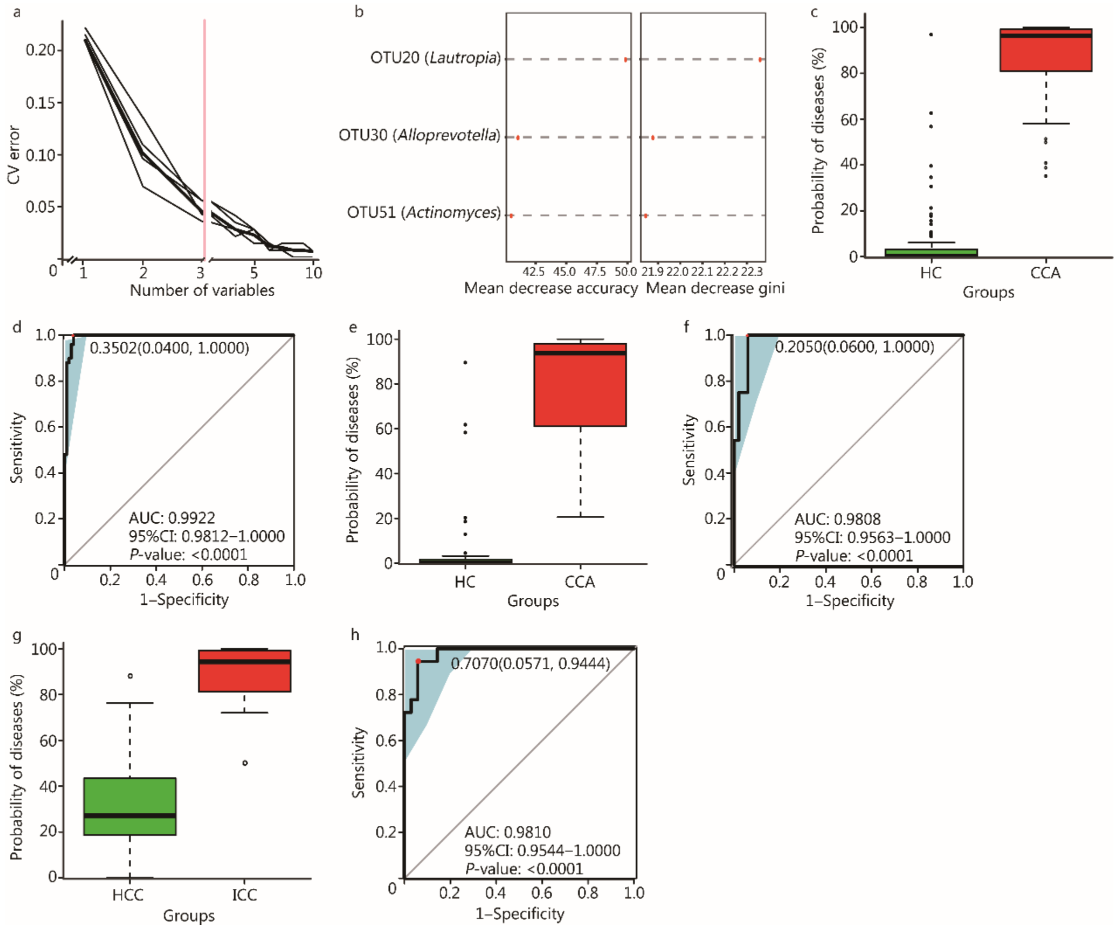


**Fig. S7 Noninvasive diagnostic model for CCA based on the oral microbiome. a** Three OUT markers [OTU20 (*Lautropia*), OTU30 (*Alloprevotella*) and OTU51 (*Actinomyces*)] were selected as the optimal marker set. **b** The mean decrease accuracy and mean decrease gini of the three markers. **c** In the discovery phase, the POD index was significantly increased in CCA group compared with HC group [0.9643 (0.8126, 0.9913) vs. 0.0027 (0, 0.0312), $P < 0.001$]. **d** The POD index achieved an AUC value of 0.9922 with a specificity of 96.00% and a sensitivity of 100.00% between CCA and HC groups. **e** In the validation phase, the POD value was significantly increased in the CCA group [0.9365 (0.6273, 0.9785) vs. 0.0020 (0, 0.0165), $P < 0.001$]. **f** The AUC value was 0.9808 with a specificity of 94.00% and a sensitivity of 100.00% in the validation phase. **g** The POD index was significantly increased in the ICC group compared with HCC group (0.9425 (0.8300, 0.9890) vs. 0.2710 (0.1860, 0.4345), $P < 0.001$). **h** The POD index achieved an AUC value of 0.9810 with a specificity of 94.29% and a sensitivity of 94.44%. OTUs operational taxonomy units, CV error, cross-validation error; POD probability of disease, CI confidence interval, AUC area under the curve, CCA cholangiocarcinoma, HC healthy control, ICC intrahepatic cholangiocarcinoma, HCC hepatocellular carcinoma
